# Molecular dependence of hippocampal bidirectional plasticity

**DOI:** 10.1186/1471-2202-15-S1-P204

**Published:** 2014-07-21

**Authors:** Joanna Jędrzejewska-Szmek, Andrew Chay, Kim T Blackwell

**Affiliations:** 1 The Krasnow Institute for Advanced Study, George Mason University, Fairfax, VA 22030, USA

## 

Hippocampal plasticity inducing protocols vary in their activation of signal transduction pathways and resulting dependence on signaling molecules. Many long-term potentiation (LTP) inducing paradigms require both memory kinases: calcium-calmodulin dependent protein kinase II (CaMKII) and protein kinase A (PKA) signaling pathways.

Both CaMKII and PKA are activated by calcium bound calmodulin, CaMKII directly, and PKA indirectly via adenylyl cyclase production of cAMP. The adenylyl cyclase is also activated by stimulation of beta-adrenergic receptor (βAR). Curiously, not all forms of cAMP dependent LTP require PKA. Specifically, LTP induced with isoproterenol (ISO) bath application followed by 100 Hz stimulation is PKA independent [1,2], whereas both ISO followed by 3 min of 5 Hz [2] and four trains of 100 Hz stimulation do require PKA [3]. Furthermore, recent experiments suggest a novel βAR signaling pathway, in which PKA phosphorylation prevents activation of G protein subtype s (Gs), and switches the βAR to the Gi subtype of G [4], leading to recruitment of another memory kinase: extracellular signal-regulated kinase (ERK).

To investigate how temporal pattern of synaptic activation determines which signaling pathways are activated, we employed a multi-compartmental stochastic reaction-diffusion model of calcium and cAMP activated signaling pathways. We investigated two model variants, one which took into account only βARcoupling to Gs, and the other which included the novel switching pathway. We performed simulations of four trains of 100 Hz, and isoproterenol application followed by either 1 train of 100 Hz or 3 min of 5 Hz, all of which produce long lasting LTP. We also simulated 3 stimulation paradigms which do not produce LTP, including 1 Hz stimulation which produces LTD.

Our results using the first model could not explain and predict the direction of synaptic plasticity based only on CaMKII, PKAc and phosphatases activity, especially for paradigms that do not induce plasticity, such as bath application of isoproteronol.

Experimentally, isoproteronol does not cause LTP despite βAR activation resulting in cAMP synthesis [2], suggesting that βAR undergo PKA dependent desensitization, that changes their coupling from Gs (stimulating adenylyl cyclases and cAMP synthesis) to Gi. Gi coupling both inhibits adenylyl cyclase, limiting the elevation in cAMP in response to bath application, and provides free Giβγ, which recruits molecules leading to ERK [5]. Including PKA dependent switching from Gs to Gi resulted in a model which could explain which molecules are activated in 5 LTP induction paradigms, 1 LTD induction paradigms, and 2 induction paradigms that do not produce plasticity. The model also allowed for proposing a measure of plasticity direction. The measure took into account activation of CaMKII, PKA, Giβγ (representing ERK), Epac and influence of phosphatase activity.
